# Gut microbiome – the key to our pets’ health and happiness?

**DOI:** 10.1093/af/vfae015

**Published:** 2024-06-20

**Authors:** Jordan E Rindels, Brett R Loman

**Affiliations:** Division of Nutritional Sciences, University of Illinois at Urbana-Champaign, Urbana, IL, USA; Division of Nutritional Sciences, University of Illinois at Urbana-Champaign, Urbana, IL, USA; Department of Animal Sciences, University of Illinois at Urbana-Champaign, Urbana, IL, USA

**Keywords:** dysbiosis, metabolites, microbiome, pet health, prebiotics, probiotics

**Implications**

- Due to lifestyle similarities and sustained intimate social interaction, humans and animals experience many of the same multifactorial diseases that are influenced by the gut microbiota.
- Physiological interactions with microbial metabolites extend across the entire body to impact virtually every aspect of animal health.
- The microbiome and metabolome can serve as biomarkers of disease risk and progression, as well as a therapeutic target for managing and treating these diseases.
- Consumption of specific dietary ingredients and supplements, especially fiber, elicits microbial production of health-promoting bioactive molecules.

## Introduction

Sixty percent of people in the United States have at least one pet, with cats and dogs being the most widely owned (Applebaum et al., 2023). Historically, companion animals were housed outside, did not receive veterinary care, and were generally left to fend for themselves. However, the human–animal bond has drastically changed over the last hundred years, with pets now playing a central role in our daily lives. Shared lifestyle and physiological exposures between humans and pets have led to increased and intimate interactions. It is, therefore, not surprising that we also share many chronic diseases.

Given these commonalities between humans and pets, companion animal health is a growing area of research. Disease etiology between pets and their owners is often extremely similar, meaning that disease research in animals can be applicable to humans and vice versa. This includes rising susceptibilities to multigenetic diseases previously unheard of in the pet population (Hayward et al., 2016; Vázquez-Baeza et al., 2016). Interestingly, risk, development, and severity of these diseases are influenced by a common factor—the gut microbiome.

The gut microbiome is a community of microorganisms inhabiting the gastrointestinal tract. Microbial communities vary greatly between species and even between individuals of the same species. These differences are attributed to factors including age and environmental exposures, particularly diet. A common misconception is that microorganisms are solely disease causing. In reality, most microbes play important roles in supporting host health. In contrast to a healthy, symbiotic gut microbiome, *dysbiosis* describes an unbalanced microbiome associated with negative health effects. However, this term is overgeneralized, and it is important to understand that not all microbiome changes elicit illness. While the identities of the microbes are important, it is products of microbial metabolism, or microbial metabolites, that elicit physiological responses. Metabolites such as short-chain fatty acids (SCFA), secondary bile acids (SBA), and neurotransmitters play a myriad of roles in mammalian physiology including programming of metabolism, immune activation, and neurotransmission. Therefore, a diverse gut microbiome is generally considered to be healthy due to metabolic flexibility and resilience to drastic change, while a less diverse microbiota may be associated with illness for opposite reasons. However, what is normal or healthy for each individual in a given population differs due to their environmental exposures, making microbiome diversity alone an inadequate measure of health status.

Despite differences in gut microbiomes between individuals, certain microbes confer health benefits. Concordantly, these species can be supplemented as probiotics. Probiotics are defined as live microbes that confer a defined health benefit to the host. Growth of commensal organisms in the gut can also be promoted by consuming dietary fiber in the form of a prebiotic (substrate that specifically feeds beneficial microbes to confer a health benefit), or as a synbiotic combination of both prebiotics and probiotics. Alternatively, fecal microbiota transplantation (FMT) involves transferring fecal microbes from a healthy individual into another individual with the intention of treating chronic disease (Chaitman et al., 2020). Although studies demonstrate positive outcomes for these interventions in very specific conditions, more work is needed to understand their therapeutic potential across diseases.

There are several important considerations while reviewing microbiome literature. Differences in the enteric microbiome between cats and dogs means that study outcomes of one species cannot be generalized to the other without validation. Dogs are generally better understood than cats, partly because Beagles are one of the most widely studied lab animals. Additionally, a major limitation of many microbiome studies is the inability to demonstrate causality between microbiome composition and disease pathogenesis. These factors mean that, in many contexts, additional research is required to comprehensively define how the microbiome influences health.

**Figure 1.**
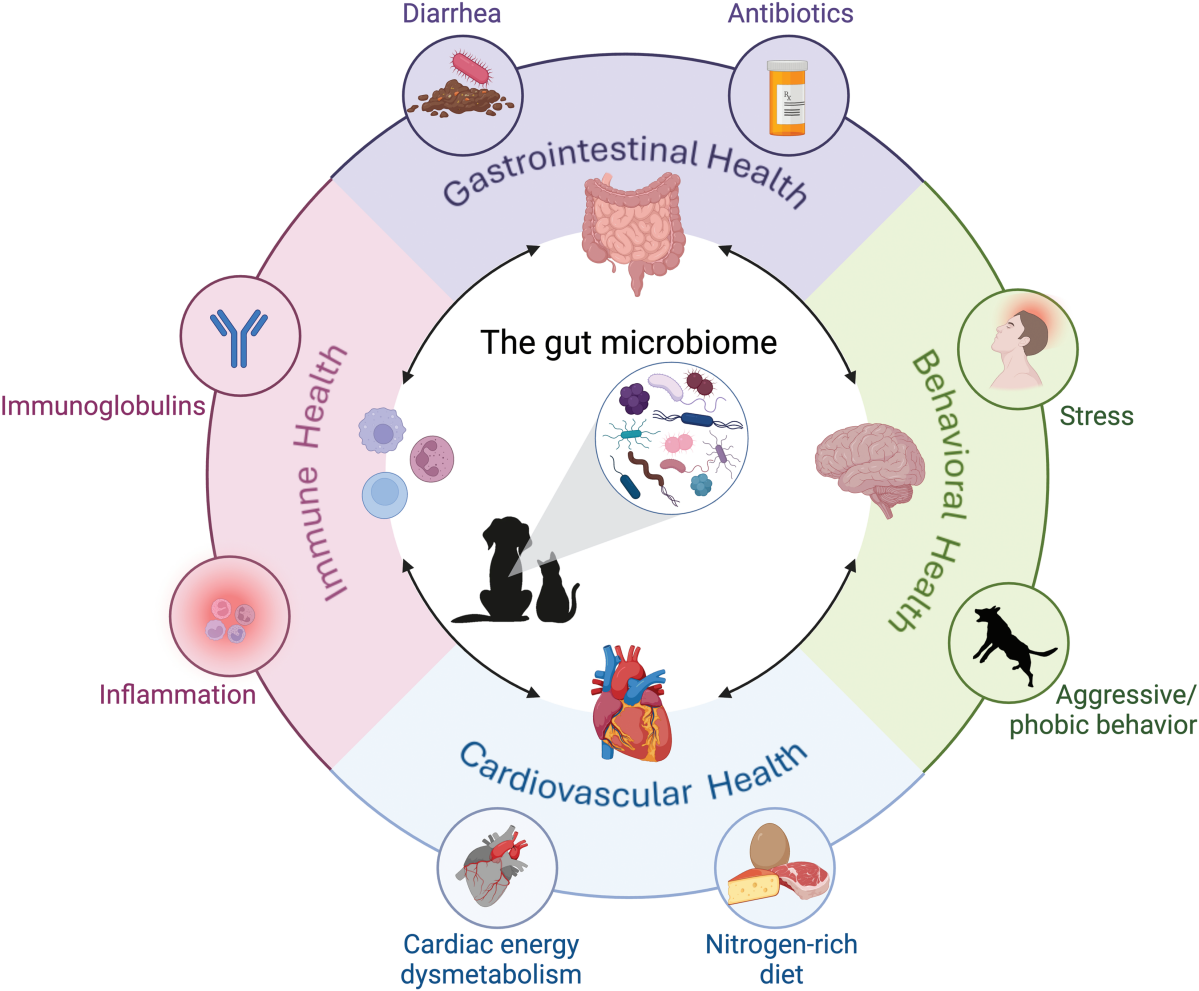
The role of the gut microbiome in systemic health. Canine and feline wellbeing is the sum health of all bodily systems (inner circle). Although these systems are anatomically distinct, intricate physiological interactions connect them all (black arrows). Systemic health is impacted by a variety of factors (outer circle) including stress, immune status, diet, and medication use. At the core of this intricate network exists a common factor—the gut microbiome (center), which plays a profound role in modulating companion animal health. Created with BioRender.com.

This review explores how the gut microbiome influences disease etiology and systemic health. Its brief nature does not allow for comprehensive review of host physiology and microbiota-mediated physiological changes and mechanisms underlying disease, which are reviewed elsewhere (Suchodolski, 2022; Homer, 2023; Stavroulaki, 2023). This review highlights diverse influences of the gut microbiome on gastrointestinal, behavioral, cardiovascular, and immune health in cats and dogs (Figure 1) as these health outcomes have the strongest evidence to indicate a role of the microbiome. We will first look at local effects within the gut itself. We will leverage this understanding of “local” health to expand our discussion to how those changes extend to and influence behavior, immune function, and cardiovascular health. Lastly, we will examine how the microbiome represents a novel target in disease prevention and treatment.

## Influence of the microbiome on gastrointestinal health

**Figure 2.**
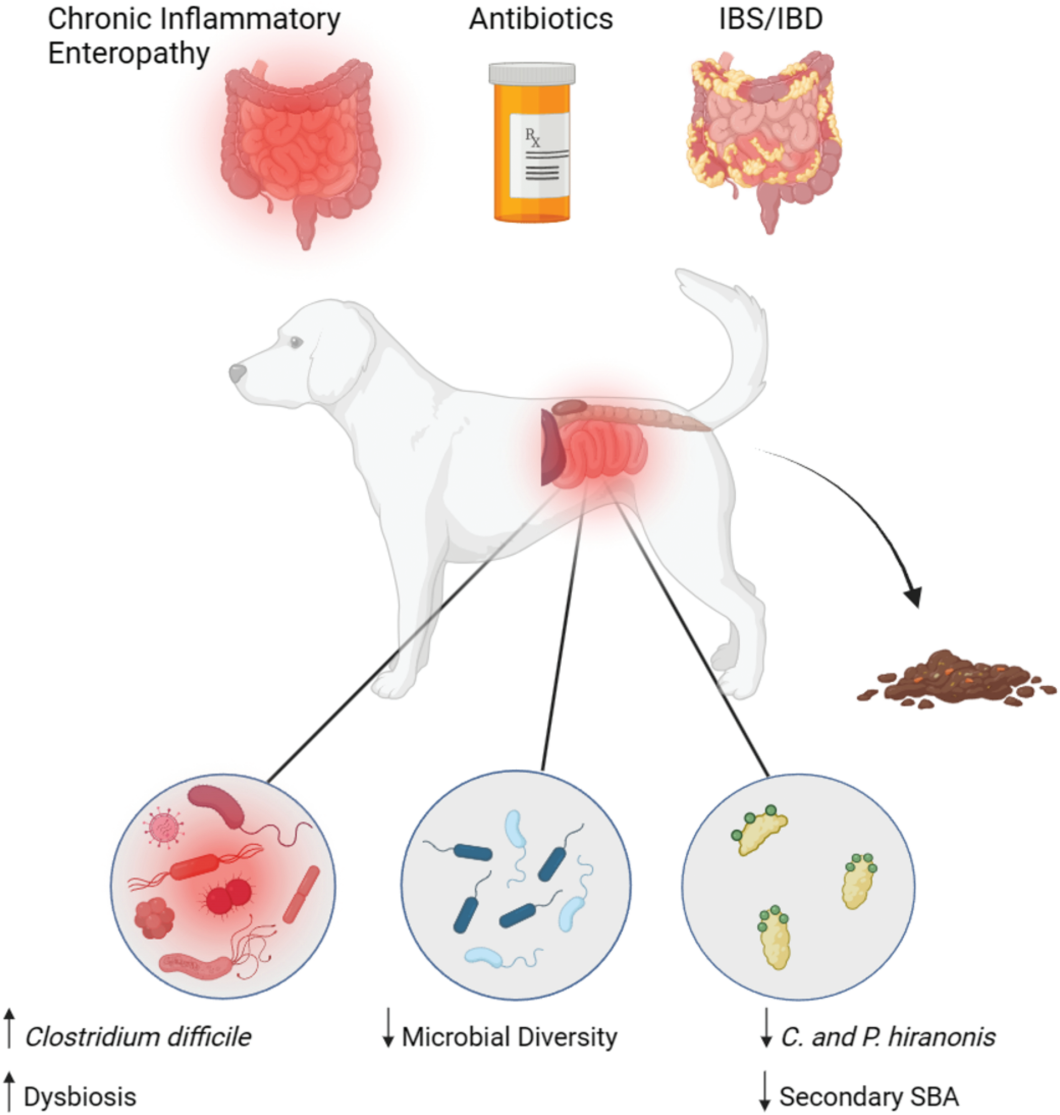
Specific microbial species and diseases are linked to diarrhea. Chronic inflammatory enteropathy, antibiotic use, irritable bowel syndrome (IBS), and inflammatory bowel disease (IBD) are associated with significant changes to the gut microbiome linked to canine diarrhea. Overgrowth of *C. difficile* causes debilitating diarrhea and gut dysbiosis (left). By contrast, *C. hiranonis and P. hiranonis* reduce diarrhea via their ability to convert primary bile acids to secondary bile acids (right). Microbiome population dynamics are complex, and the roles of most individual microbes are poorly understood. Since the microbiome is a highly interconnected network of species working together, lower microbial diversity is associated with diarrhea, while higher microbial diversity can confer resilience to diarrhea (middle). Created with BioRender.com.

While many gastrointestinal conditions affect companion animals, diarrhea is a primary concern of pet owners and leading reason for veterinary visits. Causes underlying diarrhea are multifactorial, including microbial metabolite dysregulation, medications, and preexisting conditions (Figure 2). This section highlights how bile acid dysregulation, antibiotics, and gastrointestinal diseases influence diarrhea in companion animals. Potential effective treatment options for multicausal diarrhea including implementation of microbiota-targeted interventions are discussed.

Diarrhea significantly alters the gut microbiome and metabolome (collection of all metabolites). To this end, a dysbiosis index was developed for dogs to quantify deviation from a healthy microbiome (AlShawaqfeh et al., 2017). However, a dysbiosis index may overgeneralize roles of microorganisms and attribute causation when the relationship between a microbe and disease may be purely correlative. For example, higher *Clostridium difficile* might indicate dysbiosis since infections cause debilitating gastrointestinal symptoms in pets and humans (AlShawaqfeh et al., 2017). However, low levels of *C. difficile* exist in the gut of some healthy individuals, indicating they may serve a role in the commensal microbiome that is not yet elucidated (Tian et al., 2016). As a more nuanced approach, it may be better to examine the associations between alterations in the metabolome, given that specific microbial metabolites can either exacerbate or ameliorate health conditions, and production of such metabolites can be widespread or discrete within and between microbiomes.

Secondary bile acids (SBA) are the product of microbial metabolism of primary, animal-produced, bile acids. The importance of bacterial SBA is highlighted in human studies, in which patients with irritable bowel syndrome (IBS) excrete more primary bile acids in feces, potentially linking decreased SBA to diarrhea (Vijayvargiya et al., 2019). SBA may reduce chronic diarrhea in dogs, in that dogs with diarrhea had lower abundance of *C. hiranonis,* a major contributor to SBA synthesis (Chaitman et al., 2020). Bile acid dysregulation also occurs in dogs with chronic enteropathy, a disease modeling inflammatory bowel disease (IBD) in humans. More frequent intestinal contractions, lower expression of bile acid transporters, and less SBA-producing microorganisms such as *Clostridium hiranonis* and *Peptacetobacter hiranonis* all contribute to bile acid dysregulation in pets with chronic enteropathy (Guard et al., 2019). Interestingly, cats with chronic enteropathy also experience bile acid dysregulation (Sung et al., 2023), indicating that modulation of SBA-producing microorganisms may be an effective target for treating multicausal diarrhea across species.

Veterinarians commonly prescribe antibiotics to treat pathogen-induced diarrhea. However, recent studies indicate that antibiotics are not only ineffective at treating diarrhea, but often exacerbate the condition (Manchester et al., 2019; Chaitman et al., 2020; Stavroulaki et al., 2021). Antibiotics have many side effects that persist after cessation of treatment, including antibiotic-associated gastrointestinal symptoms (AAGS) such as diarrhea, anorexia, and vomiting (Stokes et al., 2017; Manchester et al., 2019). A recent study in healthy dogs demonstrated that fecal primary bile acids were elevated for 2 months after a 7-day tylosin antibiotic treatment (Manchester et al., 2019). Additionally, administration of antibiotics decreases the abundance of *C. hiranonis* in both cats and dogs (Chaitman et al., 2020; Stavroulaki et al., 2021), indicating that antibiotics may exacerbate the underlying pathophysiology inducing diarrhea. Antibiotics should, therefore, be carefully prescribed on a case-by-case basis rather than a default treatment for diarrhea. Moreover, current research demonstrates that alternative treatments for multicausal diarrhea are on the horizon.

By contrast, supplementation of microbiota represents promising, effective new treatment options for diarrhea. A recent study found that FMT improved bile acid metabolism and increased *C. hiranonis* abundance after development of antibiotic-induced diarrhea(Chaitman et al., 2020). FMT studies in humans show that the procedure can be used to treat a variety of conditions including *C. difficile* infection, diarrhea, and multiple IBS comorbidities including depression and anxiety (Lin et al., 2021). It should be noted that despite the effectiveness of FMT, the procedure is still experimental at this time.

An alternative, more accessible, method of modulating the gut microbiome involves administration of multistrain probiotics and synbiotics. Stokes et al. studied cats which were administered antibiotics and consequentially developed AAGS. After 3 weeks of antibiotic treatment, oral administration of a multistrain synbiotic (Proviable-DC) improved food intake and decreased the vomiting frequency, but had no effect on diarrhea (Stokes et al., 2017). In a parallel experiment conducted in dogs, antibiotic administration in combination with oral synbiotic (Proviable-DC and Mycequin) mitigated the severity of diarrhea compared to placebo (Whittemore et al., 2019). Further work in this area is warranted to better understand the impact of oral synbiotic administration on gastrointestinal diseases.

Since a multitude of factors contribute to the development of diarrhea, identifying effective treatments can be challenging. Antibiotics can exacerbate symptoms, so treatments that modulate the microbiome to improve the underlying etiology of diarrhea represent promising new strategies to effectively mitigate gastrointestinal conditions while avoiding unnecessary side effects.

## Influence of the gut microbiome on behavioral health

Behavioral issues are the leading reason that animals are surrendered to shelters. Training is the most effective means of behavioral modification; however, effective training can be particularly difficult if an owner is inexperienced, too busy, or if their animal is predisposed to behavioral conditions such as anxiety or reactivity. Therefore, alternative, easily implementable ways to improve animals’ behavior are warranted. Recent research about the gut-brain axis indicates that pets’ diets may significantly influence behavior through modulation of the gut microbiome (Kubinyi et al., 2020). Specialized pet diets are already widely available in the pet food market, and behavior-focused additives are growing in popularity. This section will discuss the potential associations between microbiome composition and metabolism that influence behaviors including aggression and phobia, memory, and depression and anxiety.

Aggression is one of the most concerning pet misbehaviors, as it poses a threat to both humans and other animals. It is speculated that imbalances in hormones and neurotransmitters may contribute to aggression, but inconclusive evidence has led researchers to explore other areas, particularly the gut microbiome and their metabolites. Unfortunately, this area of research is marked by conflicting results in terms of microbes associated with behaviors. In a study by Mondo et al. (2020), microbiome profiling found that phobic dogs had elevated levels of *Lactobacillus*, a microorganism known to regulate behavior by aiding in the production of the inhibitory neurotransmitter gamma-aminobutyric acid and modifying immune function. Conversely, a parallel study by Kirchoff demonstrated that 25 different taxa of *Lactobacillus* were higher in aggressive vs. phobic dogs. Similar, contrary results are found for *Turicibacter*, a microbe responsive to the neurotransmitter serotonin. Many *Turicibacter* species are higher in aggressive dogs, while other *Turicibacter* species are higher in non-aggressive dogs (Kirchoff et al., 2019). This variability highlights a limitation of using sequencing data alone, and how the inclusion of microbial metabolites can provide a clearer picture given that microbial function varies drastically even within a single genus.

Memory allows animals to regulate their emotions and actions by recalling behaviors that were previously defined as acceptable, unacceptable, rewarding, or discouraging. In a study by Kubinyi et al. (2020), dogs with poorer memory test performances had higher relative abundance of Actinobacteria. Studies with human subjects demonstrate a positive correlation between Actinobacteria relative abundance and Alzheimer’s Disease. However, granted that Actinobacteria is a large phylum of bacteria, it is difficult to postulate what metabolic processes guide associations between these microbes and memory.

Behavioral conditions such as depression, anxiety, and aggression are complex and multifactorial. While training remains an effective strategy to improve an animal’s behavior, ongoing research suggests that certain microbial taxa may have a greater influence. Ongoing research indicates that probiotics, prebiotics, or other therapies that influence the microbiome may be effective methods of promoting behavioral health in companion animals. However, much more data are required to define the efficacy of such interventions.

## Influence of the gut microbiome on cardiovascular health

Cardiovascular diseases such as myxomatous mitral valve disease (MMVD) impact the lifespan of humans and animals, particularly when combined with obesity or old age. Altered energy metabolism, particularly energy deprivation within the myocardium, contributes to heart disease progression (Li et al., 2021b). Multiple microbial metabolites, including trimethylamine N-oxide (TMAO), SBA, and SCFA play a role in regulating cardiac function, indicating that the gut metabolome may be able to regulate the development of cardiovascular diseases. In this section, we will examine how microbial metabolites TMAO, SBA, and SCFA influence energy metabolism and management of MMVD.

Trimethylamine (TMA) is a microbial metabolite produced from the bacterial metabolism of nitrogenous compounds such as carnitine, phosphatidylcholine, betaine, and l-carnitine, which are found in high amounts in meat, dairy, fish, and eggs (Karlin et al., 2019). Following absorption, TMA is metabolized to the toxic compound TMAO, which is linked to MMVD severity. Interestingly, TMAO levels are positively associated with plasma TMAO-precursors and *E. coli* abundance (microbes capable of generating TMA) in humans and dogs with MMVD (Karlin et al., 2019; Li et al., 2021a). These associations indicate that reducing nitrogenous precursors in the diet may serve as a useful target for MMVD treatment.

SBA’s regulate energy metabolism across the body, including the cardiovascular system. Dogs with severe MMVD have a lower concentration of *C hiranonis* compared to healthy counterparts (Li et al., 2021a). Depletion of *C. hiranonis* is linked to decreased SBA, as discussed in the “Gastrointestinal Health” section. In addition to supporting energy metabolism, SBA’s inhibit *E. coli* whose association with MMVD was just discussed (Karlin et al., 2019; Li et al., 2021a). Therefore, increasing SBA may be a multifunctional target for reducing the development and progression of MMVD.

SCFA, end products of microbial fiber fermentation, may play an important role in regulating MMVD. Compared to healthy dogs, dogs with MMVD have a lower abundance of SCFA-producing microbes. Despite lower abundance of these microorganisms, total fecal SCFA concentration was not different between groups (Li et al., 2021a). Conversely, low levels of these microbes may be a consequence, rather than a cause of cardiovascular disease, meaning that the microbiome shift occurs after the onset of MMVD. Nonetheless, increasing SCFA-producing species may be a therapeutic target for MMVD.

Overall, multiple microbial metabolites influence every stage of MMVD from asymptomatic to congestive heart failure. While further work is required to define causality between activities of the microbiome and MMVD, dietary strategies to increase microbial SBA and SCFA, while limiting TMA and TMAO may prove beneficial.

## Influence of the gut microbiome on immune health

While the immune system is often associated with infectious disease, it is constantly surveying the body’s environments and cross-talking with other organ systems. Moreover, the immune system is highly involved in the health of all organs, including the gut. IBD is one noninfectious condition that afflicts us and our pets at an increasing rate. Humans and dogs with IBD have significantly altered gut microbiomes, reciprocally influencing immune responses (Soontararak et al., 2019). These stark differences between microbiomes of healthy vs. IBD individuals suggest the potential to target IBD through modulation of the gut microbiome. In this section, we will discuss the differences between immune activation in healthy dogs vs. those with IBD. Then we will discuss how stressors mimic IBD in terms of intestinal inflammation and changes to the microbiome. Lastly, we will postulate how probiotics might resolve excessive inflammation.

IBD is a chronic inflammatory condition influenced by the gut and immune system. Immunoglobulins are molecules produced by the immune system when it senses a threat, such as a pathogen. Dogs with IBD have elevated circulating immunoglobulins, generally in response to pathogenic and opportunistic microbes. Interestingly, dogs with IBD have overactive immunoglobulin responses that also target commensal and health-promoting bacteria in the gut (Soontararak et al., 2019). It is not well understood if pathogen-induced gastrointestinal inflammation precedes IBD or vice versa. Nonetheless, humans and dogs with IBD have significantly altered gut microbiomes, indicating that restoration of a balanced microbiome may have therapeutic potential.

Probiotic administration can promote a diverse and healthy microbiome while also enhancing immune function. Multistrain Slab51 probiotic has anti-inflammatory capabilities in dogs with IBD (Rossi et al., 2020). Interestingly, Pahumunto et al. (2022) demonstrated that probiotics (*L. paracasei*, *L. rhamnosus*, *L. fermentum*, *L.* rhamnosus) reduce inflammatory cytokines whether it is delivered as live cells (probiotic) or as cell wall extracts known as postbiotics. This indicates that the physical presence of the microbes, rather than just metabolic activity has anti-inflammatory capabilities

Transportation stress is a common cause of IBD-like symptoms such as diarrhea, immune activation, and intestinal permeability in cats. A study by He et al. found that antimicrobial peptide (AMP, agents that kill or neutralize microbes) administration reduce Bacteroidetes and Proteobacteria which are positively associated with gut inflammation. AMPs also reduce diarrhea and increase the abundance of *Eisenbergiella* (producers of SCFA) and *Blautia* (associated with lower inflammation; He et al., 2023). Although feline gut inflammation is an understudied area of companion animal health, the anti-inflammatory properties of AMPs warrant further investigation.

## Conclusion

Companion animal health is highly influenced by multidirectional communication between their gut microbiomes and organ systems. This review highlighted the examples of how the gut microbiome modulates gut, behavioral, cardiac, and immunological health. How these microbes and their metabolites impact health is summarized in Table 1. While complex interactions between the microbiome and their companion animal hosts are essential for regulating systemic health, mechanisms underlying these interactions require further research. Furthermore, the complexity of the gut microbiome between individuals of the same species warrants future research to address personalized responses to dietary and other microbiome-targeted treatments to ensure their maximal success. Cats are generally understudied compared to dogs, making it difficult to generalize health outcomes. Nonetheless, these studies highlight that the microbiome modulates companion animal health more than previously understood, and therefore serves as a promising therapeutic target as part of the treatment regimen of these complex diseases.

**Table 1.** An overview of the role of the gut microbiome on canine and feline health.

Species	Disease/Symptom	Underlying cause	Implicated microbes	Potential therapy	Outcomes and considerations
Dog	Diarrhea	Gut pathogens	Higher Escherichia-Shigella, *Salmonella*, *C. perfringens*, and others	Antibiotics	Can reduce healthy commensal bacteria and exacerbate symptoms, thus should only be used when deemed medically necessary.
		Antibiotic-associated gastrointestinal symptoms	Lower commensal microbiota, “Dysbiosis”	Multistrain probiotics or synbiotics	More research is needed to determine effective dosage and formulations.
		Bile acid dysregulation	Lower *C. hiranonis* or *P. hiranonis*	Enhance microbial conversion of primary bile acids to secondary bile acids	More research is needed Can be induced by antibiotic treatment
		*C. difficile* infection	*C. difficile*	Fecal microbiota transplant	More research is needed. Can be induced by chronic antibiotic use
	Inflammatory bowel disease and chronic enteropathy—diarrhea	Gut inflammation	Poorly understood	Modulation of bile acid-converting microorganisms	More research is needed Inflammatory bowel diseases are complex and multifactorial, and a variety of microbes are implicated
	Aggression, phobic behavior	Hormone and neurotransmitter imbalances	High *Lactobacillus* (phobic dogs) High *Turicibacter* (aggressive dogs)	Poorly understood	More research is needed Behavioral deficiencies are complex, involving a variety of microbes
	Memory loss	Neurodegeneration	High Actinobacteria	Poorly understood	Actinobacteria is a large phylum of bacteria, making it difficult to understand what metabolic processes are guiding the association
	Depression, neuroinflammation	Prolonged elevated cortisol	High *Escherichia-Shigella*	Prevent rapid dietary changes	More research is needed Can be onset by abrupt, drastic dietary changes Cortisol is not always pro-inflammatory
	Myxomatous mitral valve disease	Dysregulated energy metabolism	*Escherichia coli*	Reduce dietary carnitine, phosphatidylcholine, betaine, and l-carnitine, which then reduces microbial production of TMA	These nitrogenous compounds are high in meat, dairy, fish, and egg Limiting microbial TMA production reduces risk and progression of MMVD
	Inflammatory bowel disease—inflammation	Immunoglobulins	Lower commensal microbiota, “dysbiosis”	Probiotics	Probiotics have anti-inflammatory potential in dogs with IBD
Cat	Diarrhea	Gut pathogens	Higher Escherichia-Shigella, *Salmonella*, *C. perfringens*, and others	Antibiotics	Can reduce healthy commensal bacteria and exacerbate symptoms, thus should only be used when deemed medically necessary
		Bile acid dysregulation	Lower *C. hiranonis* or *P. hiranonis*	Enhance microbial conversion of primary bile acids to secondary bile acids	More research is needed Can be induced by antibiotic treatment
		*C. difficile* infection	*C. difficile*	Fecal microbiota transplant	More research is needed specifically for FMT in companion animals. Can be induced by chronic antibiotic use
	Inflammatory bowel disease and chronic enteropathy—diarrhea	Gut inflammation	Poorly understood	Poorly understood	More research is needed
	MMVD	TMAO, SBA, and SCFA	Poorly understood	Poorly understood	More research is needed
	Transportation stress	Gastrointestinal inflammation	High pro-inflammatory microbes (Bacteroidetes, proteobacteria)	Antimicrobial peptide (AMP) administration	Reduces some pro-inflammatory microbes while promoting the growth of SCFA-producing and anti-inflammatory microbes (*Eisenbergiella*, *Blautia*)

The table is organized by species, and highlights each disease, underlying cause, the implicated microbe, and potential therapy discussed in this review. Important outcomes and considerations are also addressed.

## About the Authors

**Jordan E. Rindels** graduated in May 2023 with a bachelor’s degree in animal science from Iowa State University. She is currently a graduate student in the Division of Nutritional Sciences at University of Illinois Urbana-Champaign. Her research focuses on understanding how dietary fiber can influence behavioral health by modulating the gut microbiome and its interactions with the immune and central nervous systems.

**Brett R. Loman, PhD, RD,** is an Assistant Professor in the Department of Animal Sciences and Division of Nutritional Sciences at the University of Illinois at Urbana-Champaign. His interdisciplinary research seeks to understand how dietary manipulation of the gut microbiota influences gastrointestinal physiology, and its communication with the rest of the body during health and disease. **Corresponding author:**bloman2@illinois.edu
